# Management of Patients with Nickel Hypersensitivity Undergoing Patent Foramen Ovale Closure

**DOI:** 10.3390/jcm14217540

**Published:** 2025-10-24

**Authors:** Anastasios Apostolos, Stamatios Gregoriou, Maria Drakopoulou, Georgios Trantalis, Aikaterini Tsiogka, Nikolaos Ktenopoulos, Panayotis K. Vlachakis, Paschalis Karakasis, Andreas Synetos, Georgios Tsivgoulis, Alexander Stratigos, Konstantinos Tsioufis, Konstantinos Toutouzas

**Affiliations:** 1 Unit of Structural Heart Diseases, First Department of Cardiology, National and Kapodistrian University of Athens, Hippocration General Hospital of Athens, 115 27 Athens, Greece; anastasisapostolos@gmail.com (A.A.); mdrakopoulou@hotmail.com (M.D.); geotrantalis@gmail.com (G.T.); nikosktenop@gmail.com (N.K.); vlachakispanag@gmail.com (P.K.V.); synetos@yahoo.com (A.S.); ktsioufis@gmail.com (K.T.);; 2 First Department of Cardiology, National and Kapodistrian University of Athens, Hippocration General Hospital of Athens, 115 27 Athens, Greece; 3 Department of Cardiology, Harefield Hospital, Royal Brompton and Harefield Hospitals, Guy’s and St Thomas’ NHS Foundation Trust, London SE1 9RT, UK; 4 First Department of Dermatology and Venereology, National and Kapodistrian University of Athens, Andreas Syggros Hospital, 161 21 Athens, Greece; a.tsiogka@yahoo.com; 5 Second Department of Cardiology, Medical School, Aristotle University of Thessaloniki, Hippokration General Hospital, 546 42 Thessaloniki, Greece; pakar15@hotmail.com; 6 Medical School, European University of Cyprus, 2404 Egkomi, Cyprus; 7 Second Department of Neurology, School of Medicine, National and Kapodistrian University of Athens, “Attikon” University Hospital, 124 62 Athens, Greece; 8 Department of Neurology, University of Tennessee Health Science Center, Memphis, TN 38163, USA

**Keywords:** nickel, allergy, hypersensitivity, metal, PFO, review

## Abstract

Patent foramen ovale (PFO) is implicated in cryptogenic stroke and other clinical syndromes, with transcatheter closure demonstrating superiority over medical therapy in selected patients. Most closure devices are composed of nitinol, a nickel–titanium alloy, raising concerns in individuals with nickel hypersensitivity, one of the most prevalent contact allergies worldwide. Although typically manifesting as localized dermatitis, nickel allergy has been associated with systemic reactions after device implantation, including chest pain, palpitations, migraines, dyspnea, and cutaneous eruptions. Recent evidence indicates that nickel-sensitive patients experience a significantly higher incidence of post-procedural device-related symptoms. Nevertheless, severe reactions remain rare, and the benefits of PFO closure generally outweigh the risks. The predictive value of pre-implantation patch testing remains uncertain, and the lack of nickel-free alternatives constrains device selection. Management strategies are empirical, relying on symptomatic treatment with corticosteroids, antihistamines, or device explantation in refractory cases. Future research should focus on elucidating the pathophysiology of nickel-induced hypersensitivity in cardiovascular implants, improving diagnostic algorithms, and developing biocompatible, nickel-free devices. A multidisciplinary approach involving cardiologists, dermatologists, and allergists is essential to optimize outcomes in this complex subset of patients.

## 1. Introduction

The patent foramen ovale (PFO), a remnant of fetal circulation persisting in approximately 25% of the general adult population, represents more than just a simple anatomical variation [1]. Its clinical significance lies primarily in its potential role in paradoxical embolism. Venous thromboemboli traversing this interatrial communication can bypass the pulmonary filter and enter the systemic arterial circulation [2]. This mechanism has been implicated in approximately 5% of all ischemic strokes and rises to a concerning 10% among younger stroke patients (<60 years). Notably, studies reveal that nearly half of patients aged 60 or younger presenting with an embolic stroke of undetermined source (ESUS), also termed cryptogenic stroke, have a PFO, a prevalence significantly higher than the baseline 25% observed in the general population [3,4].

The Risk of Paradoxical Embolism (RoPE) score serves as a valuable clinical tool in this context. By integrating key patient characteristics (age, history of stroke/TIA, diabetes, hypertension, smoking status) and neuroimaging findings (cortical infarct), the RoPE score estimates the probability that a discovered PFO is causally related to a cryptogenic stroke. This stratification reveals a clear gradient: patients with a low RoPE score (<3) exhibit a PFO prevalence (~23%) similar to that of the general population, suggesting a low likelihood of causality. On the other hand, patients with a high RoPE score (>8) demonstrate a markedly elevated PFO prevalence (~77%), strongly indicating a pathogenic association [4].

Robust evidence from randomized controlled trials (RCTs) supports the efficacy of transcatheter PFO closure in preventing recurrent ischemic stroke in carefully selected patients. A landmark pooled analysis of six major RCTs, encompassing 3740 patients with cryptogenic stroke and PFO, demonstrated a significant advantage for closure over medical therapy alone over a median follow-up of about six years months [5]. This represents a substantial relative risk reduction of approximately 59% and a clear indication for closure in appropriate candidates [5]. Except cryptogenic stroke, transcatheter PFO closure could also be performed in other medical conditions, such as drug-resistant migraines, platypnea–ortheodoxia syndrome, and decompression illness, but without such strong evidence [6,7,8,9].

Transcatheter PFO closure has been performed using devices structured mainly by nickel-containing alloys, predominantly nitinol, an alloy of nickel and titanium [10,11]. However, nickel cannot be considered a panacea, as it is considered one of the most frequent allergens worldwide, mainly due to causing contact dermatitis. Nevertheless, the role of nickel hypersensitivity in metal implants, endovascular devices, and PFO closure devices remains under investigation. The aim of our review is to synthesize current knowledge and address the gaps in the existing knowledge.

## 2. Nickel Hypersensitivity

Nickel hypersensitivity remains the most prevalent contact allergy globally. Ahlström et al. reported a prevalence in the European general population ranging from 8% to 19% in adults and 8% to 10% in children and adolescents, with a marked female predominance (4–10× higher than in males), driven mainly by exposure from jewelry and piercings. In dermatitis patients, the rates are increased to 12–25% in adults and 5–30% in children, reflecting heightened sensitization in those with skin barrier issues [12]. Notably, the prevalence was even higher before the EU Nickel Directive, which was implemented in 2001 and limited the quantity of nickel used. More specifically, this directive has reduced the prevalence in younger cohorts, with a significant decline in women aged 18–35 years (11.4% vs. 19.8%, *p* = 0.02) and female dermatitis patients aged ≤17 years (14.3% vs. 29.2%, *p* < 0.0001) post-regulation compared to pre-regulation periods. Similarly, dermatitis patients aged 18–30 years showed reduced rates (women: 20.2% vs. 36.6%, *p* < 0.0001; men: 4.9% vs. 6.6%, *p* < 0.0001), indicating a beneficial impact of this regulation [13]. Such findings have been confirmed by national longitudinal studies; new nickel allergy cases in adolescents dropped from 13.7% in 1995 to 11.8% in 2010 in Denmark and from 9.9% in 2000–2004 to 7.5% in 2011–2013 among Swedish adolescents [14,15,16]. However, prevalence remains high, affecting 19.8% of the general population across EU countries, with variation between countries in the most recent results [17]. The residual high prevalence might be attributed to a ‘cohort effect’, which can be observed in those older females already sensitized before the nickel regulation came into effect [18]. Additionally, the different prevalence of nickel sensitization among the countries could be associated with the date of introduction of nickel regulation; for example, higher crude estimates were found in Austria (30.32%) and Italy (26.79%), and a lower prevalence was found in Finland (14.77%) and the United Kingdom (13.1%) [19]. On the other hand, in the United States of America, Warshaw et al. documented a significant increase in nickel sensitivity from 14.3% (1994–1996) to 20.1% (2013–2014, *p* < 0.0001) among 44,097 patients patch-tested by the North American Contact Dermatitis Group, with a general population estimate around 20.1%. They highlight a female predominance (20.1% in 2013–2014 vs. 8.3% in males), with 55.5% of reactions currently clinically relevant, rising from 44.1% to 51.6% over the period (*p* < 0.0001). This is probably associated with the lack of regulation for nickel use [20].

## 3. Factors Triggering Nickel Hypersensitivity

Nickel is everywhere in the environment, ensuring unavoidable human exposure through different pathways [13]. Topical skin contact arises from metallic objects, household goods, and cosmetics, while systemic exposure may occur via food, water, surgical implants, and dental materials. The nature of nickel exposure has evolved over time, influenced by industrialization, fashion trends, and regulatory interventions [13].

Nickel-induced allergic dermatitis was initially identified as a job-related condition among male nickel platers in the 19th century, primarily affecting the hands and forearms [12]. By the mid-20th century, the focus shifted to women due to consumer goods, with workplace incidence declining due to improved safety protocols and technological advances. Despite these improvements, nickel remains a persistent issue in specific occupations [21,22,23]. Nowadays, occupational nickel allergy is prevalent in industrial environments, construction sites, and service industries including healthcare, like automotive technicians, electrical workers, and nurses [21]. Detectable nickel levels on the skin, sufficient to provoke dermatitis, have been recorded after routine tasks involving lubricants, cutting oils, tools, keys, electronic parts, coins, sewing needles, dental materials, crochet hooks, medical instruments, guitar strings, and computers [21,22,24]. The contribution of nickel as a workplace allergen can be obscured by coexisting irritants or cumulative low-dose exposures from diverse origins.

Furthermore, early records from the 1930s to 1960s linked nickel allergy and dermatitis to stocking suspenders, jewelry, eyeglass frames, and watches, with suspenders notably triggering widespread reactions. In the 1970s, metal jean buttons took precedence, followed by an earring-related surge in the 1980s that spurred regulatory action. Currently, nickel is embedded in a wide array of metallic goods and traces in detergents and cosmetics, with documented release levels eliciting dermatitis from jewelry, clothing hardware, toys, currency, tools, accessories, utensils, electronic gadgets, and eyewear [25]. While the EU Nickel Directive addresses some items (e.g., release > 0.5 μg/cm^2^/week), earrings and jewelry continue to be primary triggers [13]. Recent findings suggest that frequent brief contact with metallic objects can build nickel accumulation on the skin, potentially sustaining high allergy rates, though the significance of less conspicuous sources like cosmetics (<5 ppm in makeup) requires further investigation.

Nickel is naturally present in drinking water and various foods, creating a challenge for dietary avoidance. Notable high-nickel foods are chocolate, beans, shellfish, cereals, nuts, and canned products, with concentrations varying by soil type. Cooking equipment may further elevate nickel ingestion, as it contains nickel. Although nickel ingestion cannot initiate an allergy, it has been associated with dermatitis flare-ups and vesicular hand eczema [26]. Daily intake from a standard diet is estimated at <300 μg, with EU water limits set at 20 μg/L (typical levels: 1–10 μg/L), though a Korean case linked facial dermatitis to 53 μg/L [27]. Jensen et al. demonstrated a dose–response relationship, with 40% of nickel-allergic individuals reacting to 0.3–1 mg and 70% to 4 mg, compared to no response in controls [28].

Nickel is extensively present in dental and medical applications, contributing to unavoidable exposure in clinical settings. Dental alloys, including stainless steel used in brackets, headgear, and retention wires and nickel–chromium alloys employed in dental prostheses, are frequent. A large European investigation identified a correlation between metal allergic contact dermatitis and such dental materials. Although nickel release from these alloys is typically minimal, the corrosive oral environment is believed to enhance nickel release [29]. Evidence suggests that dental braces placed before ear piercings may induce oral tolerance, potentially reducing sensitization, though research in this domain remains limited [30].

Medical implants, mainly orthopedic and cardiovascular devices, can also release nickel and other metal ions post-insertion. Reports have documented the onset or worsening of vesicular hand eczema and dermatitis over the implant site in nickel-sensitive individuals [31]. Allergic reactions linked to surgical tools and materials have also been noted. The composition of hip replacement prostheses has evolved: metal-on-metal designs in the 1960s released significant metal particles, while metal-on-plastic variants from the 1970s produced fewer wear byproducts. In the 1980s, advanced metal-on-metal prostheses were reintroduced but are now seldom used due to patient complications [32]. An association between metal allergy and adverse implant outcomes has been proposed, though causality remains unclear [33]. Guéroult et al. showed that patch-tested nickel allergy is associated with a higher risk of adverse outcomes following endovascular device implantation [34].

## 4. Pathophysiology of Nickel Hypersensitivity

Nickel hypersensitivity, classified as a type IV delayed-type hypersensitivity reaction, represents a complex immune-mediated inflammatory response. Nickel ions, released from alloys in jewelry, dental materials, and medical devices, or even food, act as haptens that penetrate the skin, starting a cascade of immunological events [12]. Upon initial exposure, nickel activates keratinocytes, prompting the release of proinflammatory cytokines such as interleukin-1β (IL-1β) and tumor necrosis factor-alpha (TNF-α), alongside thymic stromal lymphopoietin (TSLP) [35]. These mediators stimulate antigen-presenting cells (APCs), including Langerhans cells (LCs) and dendritic cells (DCs), which upregulate major histocompatibility complex (MHC) molecules and costimulatory markers via pathways involving p38 mitogen-activated protein kinase and phosphorylated MKK6 [36]. Activated APCs migrate to draining lymph nodes, presenting nickel-haptenated peptides to naive CD4-positive T cells, leading to their differentiation into effector T cells [35].

Re-exposure to nickel triggers the elicitation phase, where recirculating hapten-specific T cells recognize the antigen, releasing inflammatory cytokines and chemokines that induce characteristic skin lesions within 48–72 h. A critical mechanism involves toll-like receptor 4 (TLR4), which nickel directly activates in human DCs, initiating nuclear factor-κB (NF-κB), p38, and interferon regulatory factor 3 signaling to amplify proinflammatory cytokine production [37,38]. This TLR4-mediated pathway distinguishes nickel from other allergens and is enhanced by lipopolysaccharide, though its role in keratinocytes may also influence wound healing via CCL5 expression [39,40]. Additionally, TSLP, produced by keratinocytes, binds to its receptor on DCs, further amplifying T cell activation and allergic inflammation, as demonstrated in mouse models where Tslp-siRNA reduced delayed-type hypersensitivity reactions. Nickel’s unique ability to directly trigger DC maturation via MKK6 and TLR4 pathways underscores its potency, with studies suggesting that inhibiting these targets (e.g., MKK6-siRNA) could mitigate hypersensitivity responses.

The pathophysiology of nickel hypersensitivity following the implantation of PFO occluders remains poorly understood, with the available literature being both limited and marked by conflicting findings, lacking a clearly documented cause–effect relationship [41]. Nickel ions, released from PFO occluders, which are composed of nitinol, a nickel–titanium alloy, elute into the bloodstream, particularly during the initial post-procedural months, potentially triggering an immune response. In vitro studies by Verma et al. provide critical evidence, showing that when these devices were submerged in Dulbecco’s phosphate-buffered saline for 90 days with measurements taken at 14 intervals, Amplatzer devices released significantly higher nickel levels compared to Gore Cardioform, which contains less exposed nickel, suggesting a device-specific variability in sensitization risk [42]. Clinically, Ries et al. were the first to investigate serum nickel levels in 67 patients before and after Amplatzer implantation, observing a peak increase at one month that remained within normal reference ranges, a trend supported by Narayana et al. in 25 patients and Elkiran et al. in 38 pediatric patients, who reported similar transient elevations without exceeding safety thresholds [43,44,45]. These findings support that nickel release likely stops after approximately 90 days, correlating with endothelialization completion.

However, this process might be disrupted in susceptible individuals by local inflammation and eosinophilia, findings observed during surgical explantation of closure devices in some case studies, suggesting a hypersensitivity reaction that could impede complete endothelial coverage [46,47]. This incomplete encapsulation may maintain nickel elution, establishing a vicious cycle of irritation and inflammation, as histological examinations of explanted tissues revealed topical eosinophilia, potentially indicative of an allergic response. We have previously proposed a mechanism involving the dendritic cells embedded in vessel walls, which could be activated upon contact with nickel ions in a way similar to their role in skin epithelial barriers, initiating the type IV delayed hypersensitivity reaction by releasing cytokines release and activating T cells [48]. Nevertheless, no study has established a direct causal link between serum nickel concentrations and hypersensitivity development, leaving the mechanism hypothetic. The presence of local inflammation potentially inhibiting endothelialization, as suggested by some reports, could sustain nickel release beyond the initial 90-day period, though this remains unproven [41]. This interplay underscores the need for longitudinal research to elucidate the role of vascular dendritic cells, quantify nickel elution kinetics, and assess their impact on hypersensitivity risk, particularly in PFO patients, in whom device-related reactions could have significant clinical implications [41].

## 5. Clinical Manifestations

Nickel hypersensitivity typically manifests as localized allergic contact dermatitis, presenting with distinct clinical patterns that vary by disease phase. In the acute phase, patients exhibit red, swollen skin often accompanied by papules, vesicles, or weeping lesions, reflecting an intense inflammatory response. On the other hand, the chronic phase is mainly characterized by dry, flaky skin, indicative of prolonged irritation and epidermal barrier disruption. Histological examination consistently reveals spongiotic dermatitis, a main finding of this condition, while significant nickel absorption may lead to secondary symmetrical rashes, suggesting systemic dissemination of the allergen. Nickel hypersensitivity is a type IV delayed hypersensitivity reaction mediated by T cell activation, which means that it does not provoke immediate systemic responses, even anaphylactic shock, in contrast with type I allergies.

In contrast, nickel hypersensitivity reactions associated with nitinol-containing devices used in PFO closure encompass a broader and more variable spectrum of clinical signs and symptoms, as summarized in Table 1. The device allergic syndrome, characterized by post-procedural chest pain, fatigue, and shortness of breath, was firstly described by Rigatelli et al. and has a significantly higher frequency in patients with confirmed nickel sensitivity compared to controls. This was evident in a study by Rigatelli et al. which included 46 patients and found that all individuals with documented nickel allergy displayed these symptoms, while the incidence was notably lower in those without allergy. Considering the subjectivity of the symptoms, this might have been a nocebo effect, associated with the fact that patients were aware of their hypersensitivity. Against this background, the Investigation of Nickel Sensitization After Percutaneous Implantation of Patent Foramen Ovale Occluder (INSPIRE) trial was an investigator-initiated, multicenter, prospective, double-blinded, randomized trial with blinded endpoint assessment, exploring whether patients with nickel hypersensitivity have a higher risk for developing device syndrome [49]. Device syndrome was defined as a composite endpoint consisting of at least one of the following: patient-reported new-onset chest pain, new-onset or worsening palpitations, new-onset or worsening migraines, dyspnea, or rash [50]. Between January 2021 and September 2024, a total of 96 patients were included in the analysis and randomized to receive either the Amplatzer PFO Occluder (n = 48) or the GSO (n = 48). All of the patients had nickel skin patch tests before the procedure, which are considered the gold standard. This analysis showed that the incidence of device syndrome was significantly higher in patients with nickel hypersensitivity compared with those without (71.4% vs. 20.6%, *p* < 0.001); this was also the case for new-onset or worsening migraines (21.4% vs. 1.5%, *p* = 0.002) and palpitations (50.0% vs. 14.7%, *p* < 0.001). No significant differences were observed in the rest of the symptoms. The important thing that eliminated the nocebo effect in the INSPIRE trial was the blinding assessment. More specifically, the endpoints were evaluated by an independent investigator who was blinded to the results of the nickel skin patch tests and device selection on a weekly basis. Patients and operators were also blinded to the nickel skin patch test results and were only informed of them after the completion of the 90-day follow-up.

According to published case reports and series, chest pain emerges as the most prevalent symptom and a primary reason for closure device explantation. Skin manifestations, such as rashes or eczematous changes, are considered classic indicators of device-related nickel hypersensitivity, constituting probably the most indicative symptom for the early recognition and diagnosis of this syndrome.

Migraines are another symptom associated with nickel hypersensitivity following PFO closure. Here, there is a paradox: PFO closure could be performed in patients with resistant migraines as a bailout treatment, but it can also cause new-onset migraines or worsen existing ones [7]. A retrospective study highlighted an increased incidence of migraines, palpitations, and chest discomfort among nickel-sensitive patients, suggesting a possible link [69]. Older studies estimated that about 15% of patients suffered from migraines following PFO closure, as seen in [70]; nevertheless, a lower incidence, about 7%, was observed in the INSPIRE trial [49]. Of note, the incidence was significantly higher in patients with than without nickel hypersensitivity; therefore, nickel hypersensitivity might play a role [71].

Furthermore, palpitations are a frequent finding in case reports and case series. Slavin et al. showed that 23% of their patients suffered from palpitations during the first six months after PFO closure, and this symptom was significantly higher in patients with nickel hypersensitivity. The INSPIRE trial showed that patients with nickel hypersensitivity suffered from palpitations significantly more frequently compared to those without (50.0% versus 14.7%, *p* < 0.001); nevertheless, the incidence of supraventricular arrhythmias and AF did not differ between patients with and without nickel hypersensitivity, but this might be attributed to the small sample size of the study [49,72]. Supraventricular tachycardias, and especially atrial fibrillation, are considered the most frequent adverse event following PFO closure. While the exact incidence varies, it is estimated to be between 2.8 and 5% [72]. The exact underlying mechanism remains unknown. Proposed pathogenetic mechanisms include local atrial irritation from the foreign occluder, triggering inflammation or scarring that disrupts anisotropic conduction and creates macro-re-entrant circuits, which is an optimal substrate for arrhythmia development [73]. Mechanical stretch from device placement, especially with bulkier first-generation devices, is implicated in AF pathogenesis, potentially altering atrial biomechanics [74]. Interference with interatrial conduction, notably via the Bachmann bundle, is suggested by increased P-wave duration post-closure, providing a substrate for arrhythmias [72]. Additionally, nickel hypersensitivity might play a role in AF development following PFO closure [75]. Before the completion of endothelialization of the device, which requires about one to three months, a notable quantity of nickel is released into the bloodstream. According to the current literature, a higher incidence of supraventricular tachycardia is reported during the first month after the procedure, which is consistent with when nickel levels increase in the bloodstream [76]. Additionally, AF usually subsides three months after closure implantation, which matches with nickel release, which stops after the endothelization of the device. Furthermore, the prevalence of nickel hypersensitivity is similar to AF incidence following PFO closure, which is about 20–25%.

Additional symptoms, including nausea, diarrhea, bronchospasm, and cardiac tamponade, have also been associated with nickel hypersensitivity in case reports and case series [41]. The transient nature of nickel release from devices, peaking within the first months before endothelialization, might contribute to these various presentations, particularly during the early post-procedural period.

## 6. Management

The predictive accuracy of pre-implantation nickel patch testing for identifying patients at risk of developing nickel hypersensitivity following PFO closure remains a subject of debate, with no consensus on its mandatory use. Kim et al. conducted a retrospective analysis to determine whether patch test results could serve as an independent predictor of post-ASD occlusion complications, finding no significant association among the 38 patients studied [77]. In contrast, Reddy et al. performed skin testing on individuals with suspected nickel sensitivity, identifying six positive cases; these patients received the Gore Helex device, which releases fewer nickel ions into the bloodstream, and none exhibited allergic symptoms post-procedure. So, this study proposed an algorithm utilizing pre-procedural nickel skin patch tests for eliminating the risk of severe allergic reactions [78]. Conversely, Rigatelli et al. identified nine patients with nickel allergy via TRUE testing, all of whom underwent transcatheter interatrial shunt closure, with eight developing device syndrome (characterized by chest discomfort, dyspnea, and weakness) in the early post-procedural period [50]. Supporting Rigatelli’s observations, Slavin suggested that pre-implantation testing may forecast allergy-related symptoms, noting a higher incidence of transient issues in nickel-sensitive individuals [50]. As the INSPIRE trial showed, the incidence of device syndrome was significantly higher in patients with nickel hypersensitivity compared to those without. Interestingly, no differences were observed between the two devices, despite the lower nickel content of the Gore Septal occluder [49]. Additionally, a subgroup analysis of the INSPIRE trial showed that device syndrome was significantly higher in female patients with than without nickel hypersensitivity, but not in male patients, recommending that nickel skin patch testing might be more useful in female than in male patients [79].

The American Contact Dermatitis Society advises against routine skin testing for all patients scheduled for surgical or transcatheter procedures, citing limited evidence [80]. Recently, a European expert consensus on characteristics of metal allergy reactions and the role of various diagnostic tools in suspected metal implant allergy was published [81]. Although it was focused on orthopedic implants, its recommendations are against allergy screening of patients without a history of potential allergy to implant components and in favor of screening of patients with a history of potential severe allergy to implant components, and there was disagreement regarding patients with potential allergy to implant components. Unfortunately, there was no discussion of PFO closure devices [82].

The primary challenge with PFO closure devices lies in the lack of alternative options. Unlike orthopedic implants, which offer devices constructed with reduced or alternative metals, PFO closure devices are uniformly made from nitinol. Although Gore devices contain less nickel, it has not been proven that they are safer for patients with nickel hypersensitivity [49,83]. Given the clinical benefits of PFO closure, the absence of alternative devices, the lack of effective treatments for patients with nickel hypersensitivity who have these implants, and the rarity of severe allergic reactions, it remains uncertain whether pre-procedural nickel skin patch tests can effectively guide clinical practice. It would undoubtedly be advantageous to have the ability to choose between current devices and novel, nickel-free alternatives, to provide specific treatments for these patients, or to reconsider the procedure if reactions were consistently severe. Against this background, the main alternative to double-disk occluders is the NobelStitch EL (HeartStitch), a percutaneous suture-based system that leaves no device within the heart [84]. Introduced in 2011, this method addresses several limitations of double-disk devices, including the need for continuous echocardiographic guidance, the risk of device migration or embolization, and patient unwillingness for permanent intracardiac device [84]. Observational studies have suggested that the NobelStitch EL is feasible and safe, yet evidence remains limited to non-controlled cohorts, and up to now, no randomized trials have been conducted [85]. As a result, its efficacy in preventing PFO-related events is still unproven. The technique appears most effective in anatomically simple PFOs, making careful patient selection critical [85]. Reported complications include partial stitch detachment, suture thrombosis, atrial tears, and knot embolisation. Although refinements have improved technical aspects over time, the procedure remains complex and highly operator-dependent [84,85]. Considering that it is a metal-free device, NobelStitch EL could be an option for patients with nickel hypersensitivity, but its lower safety and efficacy compared to conventional devices should be taken into consideration. Recently, a biodegradable device was compared with a nitinol device in a randomized study [86]. This novel device is primarily composed of a support frame, flow-blocking membrane, suture thread, shape ring, and shape thread [86]. The frame is constructed from biodegradable polydioxanone monofilament and is designed as a dual-disk structure, comprising left and right disks. A biodegradable poly-L-lactic acid flow-blocking membrane is placed inside the frame. Both materials are completely biodegradable with favorable biocompatibility. This randomized trial showed that the novel, nickel-free, biodegradable device, which disappears during the 24 months after PFO closure, showed noninferiority to a conventional nitinol PFO occluder in terms of efficacy and safety [86]. Therefore, this will an option for patients with nickel hypersensitivity when it is commercially available [86].

An option that could be viable but is unfeasible is desensitization of such patients. Up to date, there is no consensus regarding efficient nickel desensitization, as studies on oral desensitization or tolerance induction therapy have failed. Considering the lack of nickel-free devices, we should perform PFO closure in patients with nickel hypersensitivity and manage them medically in case of any adverse events. Topical corticosteroids and calcineurin inhibitors relieve the intensity and persistence of nickel dermatitis manifestations and serve as the initial therapeutic option in cases of patients with skin symptoms [12]. There is no consensus regarding the management of the remaining symptoms that could be associated with nickel hypersensitivity. We believe that the treatment should be twofold: symptomatic and causal. More specifically, we would treat patients with chest pain with analgesics, patients with migraines with anti-migraine drugs, and those with palpitations or arrhythmias with beta-blockers or each treatment recommended. Additionally, we would recommend treating with corticosteroids or antihistamines, without any clear evidence but based on case reports and our clinical experience. In case of resistant symptoms that are not relieved with medical treatment or life-threatening symptoms, surgical removal of the device and patch application on the residual shunt should be considered. Overall, only empirical therapies supported by scant evidence are employed in these scenarios; additional research evaluating the effects of various anti-inflammatory agents, such as corticosteroids, antiplatelets, and colchicine, is warranted.

## 7. Conclusions

In conclusion, while nickel hypersensitivity is a rare but clinically significant concern, the benefits of PFO closure outweigh risks in appropriately selected patients. Future directions should prioritize prospective trials to clarify diagnostic thresholds, therapeutic interventions, and innovative biomaterials. Enhanced multidisciplinary collaboration between cardiologists, dermatologists, allergists, and device manufacturers is imperative to refine risk stratification and personalize care, ultimately advancing patient safety in this evolving field.

## Figures and Tables

**Table 1:** Case reports describing patients with nickel hypersensitivity following PFO closure.

Authors	Year	Gender	Age (Years)	Closure Indication	Device	Pre-Implantation Test	Symptom Initiation	Type of Symptoms	Post-Implantation Test	Drug Treatment	Surgical Explantation	Outcome and Follow-up
Fukahara et al. [51]	2003	Female	37	PFO	Amplatzer	None	After 2 months	Dyspnea, low fever, edema	Yes, positive	None	Yes	Asymptomatic, 12 months later
Dasika et al. [52]	2003	Male	11	ASD	Gore Helex	None	Not reported	Yes, but NR	Yes, positive	None	Yes	Asymptomatic
Singh et al. [53]	2004	Female	54	ASD	Cardioseal	Yes, with device	None	None	None	None	None	Asymptomatic
Lai et al. [54]	2005	Male	38	PFO	Amplatzer	None	After several days	Chest pain, migraines, pericardial effusion	Yes, positive	Prednisolone	None	Asymptomatic, 3 weeks post-treatment
Kim et al. [55]	2008	Female	31	PDA	Amplatzer	None	After 24 h	Generalized pruritic erythematous maculopapular skin eruption	Yes, positive	Prednisolone	None	Asymptomatic, 3 months post-treatment
Rabkin et al. [56]	2009	Female	52	PFO	Amplatzer	None	After 18 months	Chest pain	Yes, positive	None	Yes	Asymptomatic, 6 months post-treatment
Belohlavek et al. [57]	2013	Female	40	PFO	Amplatzer	None	After 3 days	Atopic dermatitis, exanthema	Yes, positive	None	Yes	Asymptomatic, 12 months post-treatment
Jain et al. [58]	2013	Female	66	ASD	Amplatzer	None	After 6 months	Diarrhea, vomiting, headaches, cardiac tamponade	None	Prednisolone	No	Death at 3 months post-treatment
Prestipino et al. [59]	2014	Female	49	PFO	Amplatzer	None	After 3 months	Chest pain, migraines	Yes, positive	Corticosteroid	Yes	Asymptomatic, 6 months post-treatment
Ari et al. [60]	2017	Female	28	ASD	Lifetech Cera	Yes, with device	None	None	None	None	None	NA
Dickison et al. [61]	2018	Female	41	ASD	Amplatzer	None	NR	Chest pain, migraines	Yes, positive	No	Yes	Asymptomatic, 10 months post-treatment
Fernandes et al. [62]	2019	Male	16	ASD	Amplatzer	None	After 3 days and for 6 years	Migraines	Yes, positive	Propranolol, gabapentin, sumatriptan, flumazenil	Yes	Asymptomatic, 12 months post-treatment
Resor et al. [63]	2020	Female	37	ASD	Gore Cardioform	None	After a few days	NR	Yes, positive	NR	Yes	NR
Nair et al. [64]	2021	Female	35	ASD	Figulla Flex Occlutech	None	After implantation	Headaches, waves of pain, noise/light sensitivity, chest tightness	Yes, positive	Aspirin, clopidogrel, corticosteroids (failed)	Yes	Symptomatic relief
Kocayiğit et al. [65]	2022	Female	22	ASD	Amplatzer	None	After 1 week	Widespread itching, diffuse urticarial lesions	Yes, positive	None	Yes	Asymptomatic
Gritti et al. [66]	2023	Female	8	ASD	Amplatzer	None	After 1 week	Severe headaches, pruritus, premature canities	Yes, positive	Aspirin, clopidogrel, pizotifen, prednisone	Yes	Resolution of all symptoms
Subramanian et al. [67]	2024	Female	27	ASD	Amplatzer	None	After 10 years	Severe ulcerative colitis	Yes, positive	None mentioned	Yes	Resolution
Kumar et al. [68]	2024	Female	39	PFO	Amplatzer	None	After 1 month	Severe atypical chest pain radiating to upper extremities, occasional migraines	Yes, positive	Pregabalin (discontinued post-op)	Yes	Asymptomatic, improved QOL, exercise tolerance

## Data Availability

No new data was created.

## Abbreviations

The following abbreviations are used in this manuscript:

AF	Atrial fibrillation
APC(s)	Antigen-presenting cell(s)
CANOA	Clopidogrel and Aspirin versus Aspirin Alone (trial)
DC(s)	Dendritic cell(s)
ESUS	Embolic stroke of undetermined source
IL-1β	Interleukin-1 beta
INSPIRE	Investigation of Nickel Sensitization After Percutaneous Implantation of Patent Foramen Ovale Occluder (trial)
LC(s)	Langerhans cell(s)
MHC	Major histocompatibility complex
MKK6	Mitogen-activated protein kinase kinase 6
NA	Not available/Not applicable
NF-κB	Nuclear factor kappa-light-chain-enhancer of activated B cells
PFO	Patent foramen ovale
QOL	Quality of life
RCT(s)	Randomized controlled trial(s)
RoPE	Risk of Paradoxical Embolism (score)
TLR4	Toll-like receptor 4
TNF-α	Tumor necrosis factor-alpha
TSLP	Thymic stromal lymphopoietin
